# Quality of Anthropometric Data for Child Nutrition Monitoring in India: A Comparative Assessment Using Two Rounds of the National Family Health Survey

**DOI:** 10.3390/nu18040709

**Published:** 2026-02-23

**Authors:** Laxmi Kant Dwivedi, Somnath Jana, Rupalee Singh Chauhan, Mrigesh Bhatia

**Affiliations:** 1 Department of Survey Research & Data Analytics, International Institute for Population Sciences, Govandi Station Road, Deonar, Mumbai 400088, India; laxmikdwivedi@gmail.com (L.K.D.); somnathjana443@gmail.com (S.J.); rupaleeiipsphd@gmail.com (R.S.C.); 2 Department of Health Policy, London School of Economics, London WC2A 2AE, UK

**Keywords:** anthropometric data quality, stunting, wasting, underweight, digit preference, DHS, NFHS, India

## Abstract

Background: High-quality anthropometric data are critical for accurately monitoring child nutritional outcomes and informing policy decisions, yet inconsistencies in measurement and reporting across large-scale surveys continue to challenge data reliability. Method: This research assesses the quality of height-for-age (HAZ), weight-for-age (WAZ), and weight-for-height (WHZ) z-scores based on a repeated cross-sectional analysis of two rounds of the National Family Health Survey (NFHS-4, 2015–2016 and NFHS-5, 2019–2021), examining improvements, persistent gaps, and regional disparities. We have used WHO-recommended diagnostics including digit preference, age-heaping, completeness of measurements, biologically implausible values, and distributional properties of z-scores to evaluate the plausibility of anthropometric data and generate state-level rankings to compare transitions across rounds. Results: The results indicate modest national-level improvements in data quality in NFHS-5, particularly reductions in digit preference and implausible values; however, substantial inter-state variation remains, with some states demonstrating clear progress while others continue to exhibit measurement anomalies. The completeness of date of birth improved from 99.0% in NFHS-4 to 99.9% in NFHS-5, while completeness of anthropometric measurements declined from 98.5% to 96.6%. Digit preference for height decreased from 15.2% to 14.4%, and the proportion of biologically implausible HAZ values declined from 3.4% to 2.3%. However, the standard deviation of HAZ increased from 1.77 to 1.85 and that of WHZ from 1.40 to 1.50, indicating persistent measurement variability. Transitions in HAZ rankings further reveal mixed patterns of advancement and stagnation, with regional clustering of improvements more evident in certain parts of the country. Overall, while NFHS-5 reflects progress in anthropometric data quality, key challenges persist related to inconsistent adherence to measurement protocols, variable field performance, and inadequate supervisory oversight. Conclusions: Strengthening training, standardizing procedures, and reinforcing monitoring mechanisms are essential for achieving more reliable anthropometric data, thereby enhancing the accuracy of child nutrition estimates and supporting more evidence-based policy interventions in India.

## 1. Introduction

Child malnutrition remains a critical global public health challenge with serious consequences for human development, economic growth, and population well-being [1,2]. Despite progress in some regions, undernutrition still affects millions of children, impairing their survival, growth, and cognitive potential [3]. According to UNICEF (2023), around 148 million children under five are stunted and 45 million are wasted, with most cases concentrated in low- and middle-income countries [4]. South Asia bears nearly half of this burden, and India alone accounts for more than one-third of the world’s stunted children, approximately 36% of under-five in National Family Health Survey(NFHS-5) [1]. This places India at the heart of the global nutrition agenda and highlights the importance of high-quality data to track progress and guide effective interventions [5,6]. Addressing undernutrition is also central to the United Nations Sustainable Development Goals, especially Goals 2 and 3 on ending hunger and ensuring health for all [7]. In India, child nutrition trends are tracked through the (NFHS), which has been vital in monitoring stunting, wasting, and related indicators [8]. While NFHS-4 (2015–2016) and NFHS-5 (2019–2021) show a modest decline in stunting, wasting levels have remained stagnant [8]. Similar trends were reported for NFHS-3 (2005–2006) to NFHS 4 (2015–2016) [9]. This uneven progress underscores the need for accurate and reliable anthropometric data, as poor-quality measurements can distort estimates and lead to misguided policies [10,11]. The quality of anthropometric data is defined in terms of the completeness of age and measurement information, accuracy and consistency of height and weight recording, biological plausibility and dispersion of z-scores, absence of digit preference, and adherence to age-appropriate measurement protocols, as recommended by WHO and Demographic Health Survey (DHS) guidelines.

Accurate anthropometric indicators particularly height-for-age z-scores (HAZ) and weight-for-height z-scores (WHZ) are essential to monitor child nutritional status and evaluate progress toward national and global goals [12,13]. Large-scale household surveys such as DHS and NFHS provide standardized and comparable estimates across countries and over time [14]. However, their utility depends on the accuracy and reliability of the measurements collected in the field [10]. Over the past decade, several studies have drawn attention to persistent problems in the measurement of anthropometric indicators in large-scale surveys. Common issues include digit preference in height and weight, misreporting of age, biologically implausible values, and improper adherence to measurement protocols, imprecision due to observer error, sensitive to biases in the presence of non-directional measurement errors [15,16].Even small errors in measurement or reporting can lead to significant distortions in prevalence estimates, particularly in population where the burden of undernutrition is high [3,17]. Furthermore, transitions in survey design, field protocols, or measurement tools across rounds can introduce additional inconsistencies, making it difficult to interpret observed changes in nutritional outcomes [11].

Concerns regarding the quality of anthropometric data are not unique to India. Studies based on DHS and other national nutrition surveys from sub-Saharan Africa, Southeast Asia, and Latin America have documented similar problems, including digit preference, age misreporting, excessive dispersion of z-scores, and high proportions of biologically implausible values [10,18,19]. These cross-country findings suggest that measurement and field implementation challenges are a common feature of large-scale household surveys, reinforcing the importance of systematically assessing and improving anthropometric data quality in the Indian context as well.

These data quality issues often stem from multiple points in the data collection process. At the interviewer level, inadequate training, inconsistent application of standardized protocols, poor adherence to field procedures, and survey fatigue are well-recognized sources of measurement error. Anthropometric measurement typically occurs at the end of lengthy interviews, after modules covering fertility, child health, and household characteristics, when respondents may experience fatigue and time pressure. Under such conditions, enumerators may resort to rounding off measurements, skipping difficult cases, or measuring hastily, particularly when children are uncooperative [20]. At the respondent level, misreporting of a child’s date of birth remains a major source of age error, which directly affects the calculation of z-scores [11]. Even a one-month error can substantially bias HAZ estimates among younger children. Cultural factors, low literacy, and difficulties in recalling dates also exacerbate these problems. Such errors not only affect individual measurements but also aggregate estimates, thereby compromising the validity of population-level indicators [10,21].

Another layer of complexity arises from the timing of survey implementation, an area that has received comparatively little systematic attention in the literature. Surveys like DHS and NFHS are implemented over several months in multiple phases, and field team performance may not remain constant throughout this period [22,23]. Early in the survey, enumerators may still be adjusting to field conditions despite training, whereas in the later stages, accumulated fatigue and operational pressures may degrade performance. Seasonal variations such as temperature, child illness patterns, and clothing can also affect both the measurement process and the actual anthropometric outcomes [10,13,24]. These temporal fluctuations can systematically influence data quality, leading to heterogeneity across survey phases that is not driven by real differences in child nutrition but by measurement error. Thus, accounting for the timing of survey implementation is essential for understanding and improving data quality. To assess and monitor the quality of anthropometric data, a set of well-established diagnostic indicators is often used, including completeness of date of birth, completeness of height and weight measurements, digit preference in height and age, absolute differences in HAZ by month of birth, standard deviations of z-scores, and the prevalence of biologically implausible values [10,12]. International guidelines suggest that the standard deviation of HAZ and WHZ should typically fall between 0.8 and 1.2 in high-quality data, and substantial deviations from this range usually indicate problems with measurement accuracy [25,26]. Similarly, high digit preference values and excess flagged cases reflect interviewer errors or weak field supervision [11]. Analyses of NFHS data show that while there has been some improvement over time in certain indicators such as a decline in biologically implausible values, digit preference remains high, and standard deviations for both HAZ and WHZ exceed recommended thresholds in many states [10]. These patterns point to persistent issues related to field protocols, training, supervision, and interviewer performance that continue to affect the quality of data collected in large-scale surveys.

Given the centrality of anthropometric data in shaping nutrition policies and programmes, understanding the magnitude and sources of these measurement errors is imperative [3,27]. This study focuses on examining the quality of anthropometric data in NFHS-4 (2015–2016) and NFHS-5 (2019–2021), with particular attention to the dynamics of data quality over the course of survey implementation. Unlike previous studies that have largely concentrated on cross-sectional quality assessments, this study places emphasis on the temporal dimension of data collection, exploring whether and how the timing of data collection within the survey cycle influences the quality of anthropometric measurements [20,21]. It also investigates whether interviewer learning curves, survey fatigue, and operational sequencing may help explain observed variations in quality across states and phases.

By systematically applying WHO and DHS-recommended diagnostic indicators, including digit preference, biologically implausible values, standard deviations of anthropometric z-scores, and discrepancies in month-of-birth reporting, this study provides a comprehensive assessment of data quality [10,11]. More importantly, it situates these indicators within the context of survey implementation processes to offer a deeper understanding of the mechanisms that shape data quality in large-scale household surveys. By examining both cross-sectional patterns and temporal variations in quality, the study aims to generate empirical insights that can inform the design of more robust data collection strategies, improved training and supervision protocols, and stronger monitoring systems [12,21].

The goal of this research is to assess the quality of anthropometric data on child nutritional status in India using height-for-age z-scores (HAZ) and weight-for-height z-scores (WHZ) from the fourth (2015–2016) and fifth (2019–2021) rounds of the Demographic and Health Surveys, implemented as the NFHS [8]. It examines key data quality indicators, including digit preference, biologically implausible values, completeness of measurements, and accuracy of age reporting. In addition, the study explores whether the timing of survey implementation affects data quality by analysing variations across different phases of fieldwork. This approach aims to provide a clearer understanding of the factors influencing measurement quality and to inform strategies for improving the reliability of large-scale nutrition surveys.

## 2. Materials and Methods

### 2.1. Data Source

This study draws upon unit-level data from the NFHS-4 (2015–2016) and NFHS-5 (2019–2021), Indian version of the DHS series conducted by the International Institute for Population Sciences (IIPS), Mumbai. The sample design for NFHS is a stratified sample selected in two stages. Stratification was achieved by separating each district into urban and rural areas, where villages and Census Enumeration Blocks (CEBs) was selected as Primary Sampling Units (PSUs) in the first stage. Small PSUs with fewer than 40 households (HHs) was linked to the nearest geographically located PSUs. A second stage of stratification in the rural areas was achieved based on the village size (number of HHs). The implicit stratification at PSU level was achieved by using indicator of percentage of Scheduled Castes (SC)/Scheduled Tribes (ST) population and female literacy rate. Within each rural stratum (three), in the first stage, villages were selected with probability proportional to size (PPS) sampling using census 2011 sampling frame. In the urban areas of each district, implicit stratification was achieved by sorting the sampling frame according to the percentage of SC/ST population and then selected the CEBs by using the PPS selection procedure. A household listing operation was carried out in all of the selected PSUs before the main survey. The household listing operation consists of visiting each of the selected PSUs and listing all residential HHs found in the PSU. The resulting list of households serves as the sampling frame for the selection of households in the second stage. All women aged 15–49 in the selected households were eligible for the interview, and all men aged 15–54 in the State-Module selected households were eligible for the interview.

NFHS-4 covered around 0.6 million households, 2.8 million individuals, and 0.7 million eligible women aged 15–49 years, while NFHS-5 covered a similar number of households and women, along with approximately 0.1 million men. The surveys were implemented in phases across states, engaging 14 field agencies and 789 field teams in NFHS-4, and 17 agencies with 1061 field teams in NFHS-5. Each team comprised one field supervisor, three female interviewers, one male interviewer, two health investigators, and a driver. Ethical approval for data collection was granted by the ICF Institutional Review Board and ICMR (Indian Council of Medical Research), and all survey procedures adhered to international ethical standards. In both NFHS-4 and NFHS-5, anthropometric measurements were collected by trained health investigators using standardized DHS-recommended equipment and protocols. Length/height was measured using portable stadiometers/infantometers and weight using calibrated digital weighing scales. Children aged 0–23 months were measured in the recumbent position, while children aged 24–59 months were measured standing, following WHO 2006 guidelines [25]. Field investigators received centralized training, standardization exercises were conducted before field deployment, and measurements were taken following DHS standardization [11,14,25]. The present study uses the anthropometric z-scores provided in the DHS/NFHS datasets, which are computed using the WHO Child Growth Standards [18].

### 2.2. Data Quality Indicators

The assessment of data quality in this study was based on the indicators recommended by the WHO–UNICEF Joint Working Group on Anthropometric Data Quality and supported by previous empirical research. Separate sets of indicators were constructed for Height-for-Age Z-scores (HAZ) and Weight-for-Height Z-scores (WHZ) to evaluate the precision and reliability of anthropometric measurements in both NFHS rounds. Eight indicators were used to assess HAZ, and five indicators were used to assess WHZ.

#### 2.2.1. Indicators for Height-for-Age (HAZ)

The indicators used to assess the quality of HAZ included (i) completeness of date of birth, (ii) completeness of anthropometric measurement, (iii) digit preference for height, (iv) digit preference for age, (v) absolute difference in mean HAZ by month of birth, (vi) biologically implausible or flagged values, (vii) dispersion of HAZ, and (viii) inappropriate height measurement. The completeness of the date of birth was defined as the proportion of children aged 0–59 months whose month and year of birth were reported. Missing days of birth were imputed using the WHO Anthro software. Completeness of anthropometric measurement referred to the proportion of children who had valid height and weight data recorded; incomplete records resulted from absences, refusals, or measurement errors.

Digit preference for height and age was assessed using the Index of Dissimilarity, calculated as half the sum of absolute differences between observed and expected frequencies of terminal digits. A higher value of this index indicates stronger rounding tendencies, reflecting measurement or reporting bias. The absolute difference in mean HAZ by month of birth (MOB) was computed to detect systematic misreporting of age, comparing mean HAZ values of children reported as born in January and December. A smaller difference indicates greater reliability in age reporting. Biologically implausible values were defined using WHO thresholds HAZ values beyond ±6 SD were flagged and excluded from analysis. The dispersion of HAZ, measured through standard deviation after removing flagged cases, reflected the variability in measurement; values exceeding 1.0 generally suggested data quality issues. Finally, inappropriate height measurement was determined following WHO guidelines, where children aged 6–23 months measured while standing or those older than 24 months measured lying down were categorized as incorrectly measured.

#### 2.2.2. Indicators for Weight-for-Height (WHZ)

The quality of WHZ was examined using five indicators: completeness of anthropometric measurement, digit preference for height, biologically implausible values, dispersion of WHZ, and inappropriate height measurement. Similar to the HAZ indicators, implausible WHZ values were defined as those exceeding ±5 SD of the WHO reference population. The standard deviation of WHZ was used to assess variability, with higher values indicating potential measurement errors or inconsistencies.

### 2.3. Statistical Analysis

All analyses were performed using STATA version 19 and Microsoft Excel. All anthropometric outcomes are analysed using age- and sex-standardized z-scores based on the WHO 2006 growth standards [18], which are computed in months and therefore already account for growth differences within a given year of age; the age-related quality indicators used in this study are intended to detect age misreporting rather than biological variation in growth. Each data quality indicator was first calculated at the state level for both NFHS rounds. To ensure comparability across states, the indicators were normalized using a range standardization method, which transforms all values into a common scale between 0 and 1, thereby aligning their directionality and maximizing the variance across observations.

Following normalization, Principal Component Analysis (PCA) was employed to construct composite indices representing the overall quality of anthropometric data for HAZ and WHZ separately. PCA was chosen for its ability to reduce multidimensional data into a smaller number of uncorrelated components that capture the maximum possible variance.

PCA was used to construct composite indices representing overall anthropometric data quality for HAZ and WHZ indicators. Prior to PCA, all indicators were normalized using range standardization to ensure comparability across different scales and to align their directionality. The correlation matrix of the standardized indicators was then used as the input for PCA. The number of components to be retained was determined using the Kaiser criterion, whereby only components with eigenvalues greater than 1 were kept, as these explain more variance than an individual original variable. To improve the interpretability of the retained components and to obtain a simpler and more meaningful factor structure, orthogonal varimax rotation was applied. The rotated component loadings were examined to confirm that the indicators contributed meaningfully to the underlying data quality dimension. Finally, component scores were computed and used to construct a composite data quality index, which was then employed to rank states and to compare overall anthropometric data quality across survey rounds and fieldwork phases.

To further assess the performance of field investigators, PCA was applied to evaluate the team-level data quality across selected indicators. Eight large states Bihar, Tamil Nadu, Karnataka, Andhra Pradesh, West Bengal, Uttar Pradesh, Madhya Pradesh, and Rajasthan were selected for this analysis based on their varied malnutrition burden and diversity in survey implementation contexts. Within each state, field investigators were grouped into district “sets” according to their sequence of data collection. Teams were assumed to move systematically from one set of districts to another, allowing for the assessment of performance variation across the survey cycle. The ranking of data quality indicators across successive sets was then analyzed to understand patterns of improvement, consistency, or deterioration over time, potentially reflecting the impact of training, supervision, and survey fatigue.

## 3. Results

### 3.1. Overview of Anthropometric Data Quality

The results highlight both improvements and persistent challenges in the quality of anthropometric data between NFHS-4 (2015–2016) and NFHS-5 (2019–2021). At the national level, indicators such as completeness of date of birth and anthropometric measurements remained high, while issues such as digit preference and inappropriate measurement practices continued to affect data reliability. Table 1 presents the national summary statistics for key quality indicators across the two survey rounds.

Overall, the completeness of date of birth improved marginally from 99.0% in NFHS-4 to 99.9% in NFHS-5, reflecting progress in accurate recording of basic demographic information. Completeness of anthropometric measurements slightly declined from 98.5% to 96.6%, possibly indicating operational constraints or increased respondent non-cooperation in the latter round. Digit preference for height, which indicates rounding tendencies, showed a modest improvement, decreasing from 15.2% to 14.4%, while digit preference for age also declined from 0.8% to 0.6%, suggesting reduced heaping at specific ages. The proportion of biologically implausible HAZ values declined from 3.4% to 2.3%, indicating better adherence to measurement protocols. However, the standard deviations (SDs) for HAZ and WHZ increased slightly (from 1.77 to 1.85 and 1.40 to 1.50, respectively), signalling persistent variability in measurement precision. Height inappropriateness also rose from 5.0% to 5.6%, suggesting some inconsistency in following age-specific height/length measurement procedures (Table 1).

### 3.2. State-Level Variations

Substantial heterogeneity in data quality was observed across states (Table 2 and Table 3). Most states demonstrated nearly complete reporting of date of birth and high completeness in anthropometric data. However, a few states, notably Sikkim and Delhi, showed relatively lower completeness in measurements, possibly due to logistical challenges during fieldwork. The index of dissimilarity for digit preference in height varied widely across states, ranging from 8.5% in Manipur to 22.5% in Arunachal Pradesh during NFHS-4, and from 11.3% in Rajasthan to 23.9% in Lakshadweep during NFHS-5, indicating continued variation in field measurement practices.

The distribution of biologically implausible values for both HAZ and WHZ revealed improvement in most states, yet certain northeastern and hilly states (e.g., Arunachal Pradesh, Mizoram, and Nagaland) continued to record relatively higher shares of implausible values, suggesting difficulties in measuring younger children and variations in field supervision quality. The SD of HAZ and WHZ scores also exhibited notable state-level variation. For instance, SD of HAZ ranged from 0.8 in West Bengal to 9.3 in Arunachal Pradesh in NFHS-4, and from 1.0 in Andhra Pradesh to 11.5 in Arunachal Pradesh in NFHS-5. Similarly, SD of WHZ ranged from 0.7 in Telangana and Andhra Pradesh to 7.9 in Arunachal Pradesh during NFHS-4, and from 0.8 in Andhra Pradesh to 10.1 in Lakshadweep during NFHS-5. These large deviations indicate persistent inconsistencies in anthropometric measurements in several regions despite broader national-level improvement.

### 3.3. Transitions in State-Level Rankings

**Figure 1:**
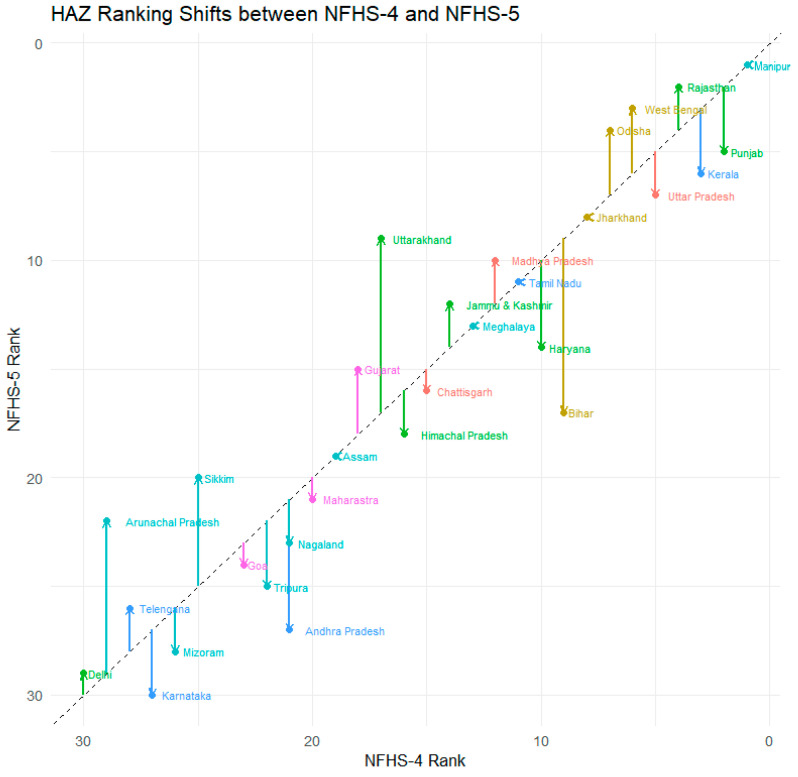
Transition in state-level HAZ rankings between NFHS-4 (2015–2016) and NFHS-5 (2019–2021).

**Figure 2:**
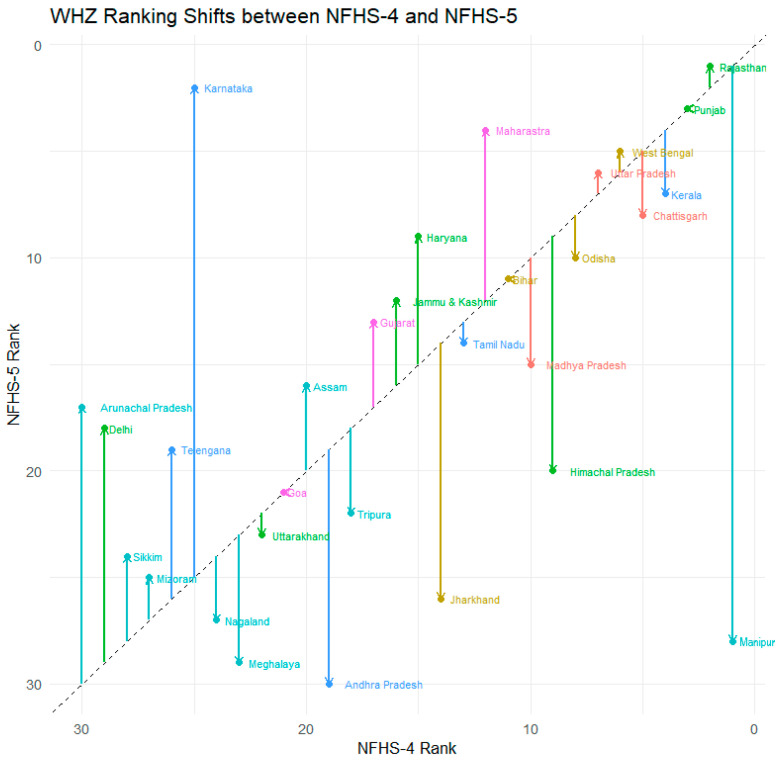
Transition in state-level WHZ rankings between NFHS-4 (2015–2016) and NFHS-5 (2019–2021).

Figure 1 and Figure 2 illustrate the transitions in state-level rankings for HAZ and WHZ data quality between NFHS-4 and NFHS-5. The comparison shows mixed trends across regions. In southern India, data quality declined slightly for Andhra Pradesh, Karnataka, and Kerala, while in the northern region, Haryana and Himachal Pradesh also showed marginal deterioration. Conversely, several states in the northeastern, central, and eastern regions demonstrated improvement in rankings, suggesting enhanced training and supervision in those areas. For WHZ, Andhra Pradesh experienced a notable decline in ranking (from 19th to 30th position), while Telangana improved significantly (from 26th to 19th position). Northern, northeastern, and central states, including Himachal Pradesh, Manipur, and Madhya Pradesh, generally improved in WHZ data quality, reflecting more consistent measurement practices in NFHS-5 (Figure 1 and Figure 2).

### 3.4. Performance of Field Investigators

**Figure 3:**
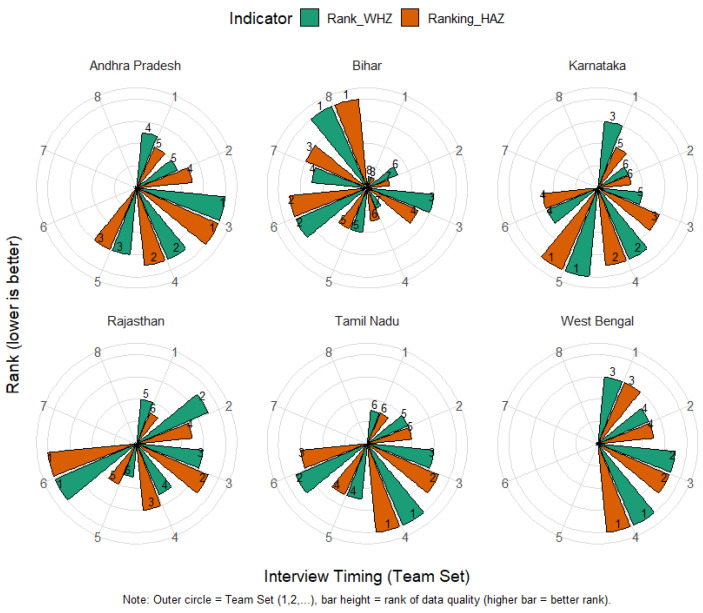
Ranking of HAZ and WHZ data quality indicators by the timing of the interview (denoted by team set) for major states of India NFHS-5 (2019–2021).

**Figure 4:**
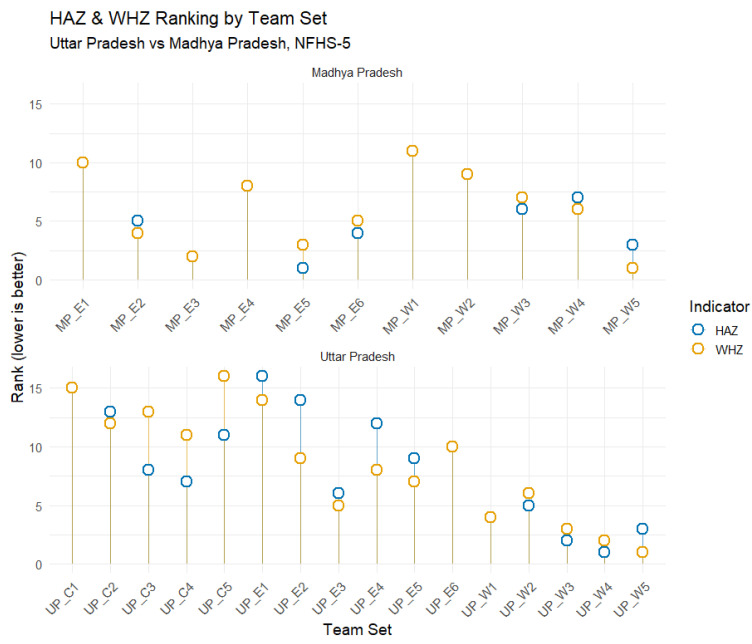
Comparative Rankings of HAZ and WHZ Across Uttar Pradesh and Madhya Pradesh Subregions from NFHS 5 (2019–2021). MP_E: Madhya Pradesh Eastern; MP_W: Madhya Pradesh Western; UP_C: Uttar Pradesh Central; UP_E: Uttar Pradesh Eastern; UP_W: Uttar Pradesh Western; The numeric suffix of each state represents the team number.

An analysis of field investigators’ performance across different phases of the survey revealed notable temporal variation. As shown in Figure 3 and Figure 4, in NFHS-5, the data quality improved steadily towards the later phases of the survey, indicating that investigators likely gained experience and efficiency over time. In contrast, during NFHS-4, the best performance was observed during the middle phase, suggesting possible learning followed by fatigue effects.

State-specific analyses revealed further variation. In Bihar and West Bengal, later field teams performed better, possibly reflecting improved supervision and cumulative field experience. In contrast, southern states such as Tamil Nadu, Karnataka, and Andhra Pradesh exhibited inconsistent trends, with only Rajasthan demonstrating sustained improvement towards the end of the survey. Regional of Uttar Pradesh and Madhya Pradesh also indicated intra-state differences, with Uttar Pradesh and Madhya Pradesh showing relatively better data quality than their eastern counterparts. These findings suggest that field experience, monitoring intensity, and agency-level management significantly influence anthropometric data quality (Figure 3 and Figure 4).

## 4. Discussion

This study provides a comprehensive assessment of the quality of anthropometric data collected in two recent rounds of India’s National Family Health Survey (NFHS), offering new insights into patterns of measurement error, investigator performance, and inter-state variability. While the findings show modest improvements in overall data plausibility between NFHS-4 (2015–2016) and NFHS-5 (2019–2021). First, although several indicators such as digit preference and the proportion of biologically implausible values show modest improvement in NFHS-5, dispersion of z-scores and height inappropriateness remain persistent concerns. Second, substantial inter-state heterogeneity in data quality continues to exist, indicating uneven implementation and supervision across regions. Third, the phase-wise analysis suggests learning effects among field investigators, with improved data quality in later phases of NFHS-5. Finally, the findings highlight that improvements in national averages can mask important subnational and operational-level weaknesses in measurement quality. These results highlight that despite progress in training and field supervision, maintaining standardized and accurate anthropometric measurements in large-scale surveys remains a major challenge.

The improvement in the completeness of demographic and anthropometric data, as well as the decline in digit preference and biologically implausible values, suggests gradual enhancement in field procedures and interviewer training. These patterns are consistent with previous studies demonstrating that systematic monitoring, the use of electronic data capture, and protocol refinement improve measurement reliability over time [3,28]. However, the marginal increase in standard deviations of both height-for-age (HAZ) and weight-for-height (WHZ) z-scores indicates residual variability in measurement quality, echoing concerns raised by Mei and Grummer-Strawn (2007), who noted that even small deviations in measurement precision can significantly bias prevalence estimates of stunting and wasting [18,29]. Unlike earlier studies that primarily focused on cross-sectional assessments of data quality, the present analysis highlights the dynamic nature of measurement quality during survey implementation and shows that both learning effects and fatigue-related patterns can coexist, depending on survey organization and monitoring intensity.

State-level analyses revealed notable heterogeneity, with several northeastern and hilly states continuing to exhibit greater data inconsistency. These findings likely reflect logistical difficulties in accessing remote terrain, variation in training implementation, and limited supervisory oversight issues also highlighted in regional assessments of DHS data quality in similar low-resource contexts [19,30]. Conversely, the observed improvement in data quality in central and eastern Indian states may reflect increased experience among survey agencies and a stronger focus on field-level monitoring. Importantly, states with historically higher literacy and health infrastructure such as Kerala and Tamil Nadu did not always record superior data quality, suggesting that technical and procedural dimensions of survey execution may outweigh background socio-economic advantages.

The analysis of field investigator performance across survey phases revealed that data quality tended to improve toward the later stages of NFHS-5, implying cumulative learning and adaptation among investigators [31]. In contrast, NFHS-4 showed higher performance during the middle phase, followed by a decline, which may point to fatigue effects, a phenomenon previously noted in multi-round surveys [32]. These temporal patterns underscore the critical role of continuous supervision, refresher training, and real-time feedback mechanisms during large-scale data collection exercises. Evidence from methodological audits of the DHS program supports this finding, showing that frequent monitoring and supportive supervision substantially improve anthropometric accuracy [17,33].

The persistence of height inappropriateness, reflected by children being measured in the wrong position relative to their age, remains a matter of methodological concern. Such procedural errors are well documented in anthropometric literature and can distort population-level estimates of stunting and wasting [5,7,11]. The higher prevalence of such errors in some states suggests the need for reinforced training on measurement protocols and the use of standardized height boards and length mats. Additionally, variability in standard deviations and flagged cases across states highlights potential inter-agency disparities, implying that the consistency of survey management practices plays a crucial role in determining data quality outcomes.

An important public health implication of this study is that several simple and easily computable indicators such as digit preference, the proportion of biologically implausible values, standard deviation of z-scores, and height inappropriateness can serve as effective real-time tools for monitoring data quality during survey implementation. Our findings show that states and survey phases with poorer performance on these indicators also exhibit greater overall measurement inconsistency, while improvements in these indicators in NFHS-5 are accompanied by better overall data quality rankings. These indicators can therefore be routinely used by survey managers and supervisors to quickly identify underperforming teams, target corrective training, and strengthen field supervision, ultimately improving the reliability of child malnutrition estimates used for public health planning and policy.

Collectively, these findings reaffirm that anthropometric data quality is not solely a function of respondent characteristics or sampling design but is strongly influenced by investigator skill, supervision intensity, and field logistics. Studies have shown that even small, systematic measurement errors can lead to over- or underestimation of malnutrition prevalence by up to five percentage points [34,35]. Therefore, improving the technical rigor of field measurement remains essential for ensuring the credibility of national nutrition estimates.

This study has several limitations. First, the analysis is based on only two rounds of NFHS (NFHS-4 and NFHS-5), which limits the ability to examine longer-term trends in anthropometric data quality. Second, although the study focuses on measurement quality, some indicators, particularly those related to age, may also reflect respondent-level recall and reporting bias that cannot be fully separated from interviewer error. Third, as this is a secondary data analysis, we are unable to directly observe field measurement practices or verify protocol adherence at the time of data collection. Finally, while the composite indices summarize multiple dimensions of data quality, some degree of simplification is inevitable when complex measurement processes are reduced to summary indicators

## 5. Conclusions

The findings of this study underscore several critical patterns in anthropometric data quality that have direct implications for interpreting national nutrition estimates. First, although NFHS-5 shows improvements in completeness, digit preference, and reductions in implausible values, the persistent elevation in the HAZ and WHZ standard deviations across many states signals ongoing inconsistencies in measurement precision. Second, the analysis reveals that state-level disparities remain substantial, with remote and northeastern states consistently exhibiting higher measurement errors, highlighting structural challenges in field implementation. Third, the study demonstrates clear investigator-learning effects, with improved data quality in later phases of NFHS-5, suggesting that experience, supervision, and operational familiarity play a crucial role in enhancing measurement reliability. Routine use of simple data quality indicators such as digit preference, flagged cases, and z-score dispersion during fieldwork can substantially strengthen the reliability of anthropometric data and, in turn, improve the evidence base for nutrition policy and programme targeting. Collectively, these results affirm that while India has made progress in anthropometric data quality, systematic efforts are still required to minimize procedural errors and inter-regional variability to ensure accurate monitoring of child malnutrition.

To strengthen future survey rounds, emphasis should be placed on three key areas: (i) continuous in-field supervision and retraining, to minimize procedural errors and ensure protocol fidelity; (ii) standardization of measurement equipment and procedures, particularly in remote and high-burden states; and (iii) team-level performance monitoring, using real-time feedback and digital quality checks to identify and correct systematic biases during data collection. Furthermore, ensuring consistency across survey agencies and establishing independent audit mechanisms can significantly enhance comparability and confidence in anthropometric indicators.

Reliable anthropometric data form the foundation for evidence-based nutrition policy and for tracking progress toward national and global goals such as the Sustainable Development Goals (SDGs). Enhancing data quality through robust methodological oversight is therefore not merely a technical exercise but a public health imperative. Strengthening anthropometric measurement systems in India’s NFHS will ensure that future estimates of stunting and wasting more accurately reflect the true nutritional landscape, enabling policymakers to target interventions with greater precision and equity.

## Figures and Tables

**Table 1:** Summary Statistics of teams’ performance in each quality indicator for HAZ, and WHZ, India, NFHS-2015–2021.

	NFHS-4 (2015–2016)	NFHS-5 (2019–2021)
**Complete DOB**	99.01	99.92
**Complete anthropometric measurements**	98.46	96.57
**Digit Preference height (%)**	15.19	14.36
**Digit Preference Age (%)**	0.78	0.62
**Implausible values HAZ**	3.41	2.28
**Implausible values WHZ**	4.70	4.20
**Absolute Difference in mean HAZ by MOB (%)**	0.28	1.92
**SD of HAZ (%)**	1.77	1.85
**SD of WHZ (%)**	1.40	1.50
**Height inappropriate**	4.99	5.60

HAZ = Height-for-Age Z-score; WHZ = Weight-for-Height Z-score; SD = Standard Deviation; MOB = Month of Birth; DOB = Date of Birth.

**Table 2:** Summary statistics of Data quality indicators for HAZ and WHZ for states of India, NFHS-4, 2015–2016.

State	Completeness of Date of Birth, %	Completeness of Anthropometry Measurement, %	Digit Preference for Height, Index of Dissimilarity, %	Digit Preference for Age at 6-Mo Intervals, Index of Dissimilarity, %	Biologically Implausible (“Flagged”) Values for HAZ, %	Biologically Implausible (“Flagged”) Values for WHZ, %	Absolute Difference in Mean HAZ by Month of Birth (December vs. January), of HAZ, *Z* Score	SD of HAZ, Z Score	SD of WHZ, *Z* Score	Height Inappropriately Measured
Andhra Pradesh	96.7	94.5	14.7	0.3	6.1	6.3	0.3	0.9	0.7	5.69
Arunachal Pradesh	96.8	92.0	22.5	0.5	10.8	12.1	0.3	9.3	7.9	5.64
Assam	99.1	96.2	16.2	0.9	5.6	6.7	0.3	2.2	1.8	4.44
Bihar	99.2	99.4	12.0	0.5	2.8	3.2	0.3	1.6	1.1	8.79
Chhattisgarh	99.8	99.7	15.7	1.0	1.3	2.3	1.3	1.9	1.7	1.86
Goa	99.7	99.6	18.2	1.4	2.3	6.9	0.8	2.6	2.1	4.45
Gujarat	97.3	96.9	15.5	0.5	4.8	7.0	0.3	1.6	1.2	5.03
Haryana	99.5	98.9	13.9	0.6	3.6	5.5	0.4	2.1	1.8	6.5
Himachal Pradesh	98.7	97.5	13.0	1.3	4.0	4.7	0.0	2.4	2.1	2.66
Jammu And Kashmir	99.7	98.4	12.8	0.9	4.5	5.5	0.2	3.7	3.0	5.67
Jharkhand	99.7	98.9	14.0	0.4	3.2	5.6	0.3	2.3	1.8	5.86
Karnataka	95.6	98.6	19.4	0.8	5.0	7.3	0.5	1.7	1.4	7.29
Kerala	99.4	98.9	9.6	0.7	3.8	5.6	0.1	1.3	1.1	4.83
Madhya Pradesh	98.8	99.4	14.5	0.7	2.6	3.6	0.3	2.1	1.6	5.19
Maharashtra	97.5	97.6	13.4	1.0	4.7	7.2	0.2	1.3	1.0	4.63
Manipur	99.8	98.9	8.5	0.4	1.6	2.4	0.0	4.8	4.1	1.79
Meghalaya	99.7	98.9	20.3	0.4	3.9	5.6	0.1	4.5	3.6	3.62
Mizoram	98.8	98.9	18.4	1.0	3.9	4.3	0.2	7.6	6.0	5.41
Nagaland	99.4	98.9	15.4	1.1	8.9	10.2	0.3	6.4	5.1	2.77
NCT of Delhi	96.0	80.1	17.8	0.3	21.0	22.2	0.6	1.2	1.0	2.31
Odisha	98.7	99.0	12.9	0.5	3.1	3.8	0.2	1.9	1.6	4.38
Punjab	99.9	99.2	12.9	0.5	2.0	3.4	0.3	1.7	1.5	1.9
Rajasthan	99.8	99.2	11.4	1.0	2.1	3.8	0.2	1.8	1.5	2.48
Sikkim	100.0	98.3	21.1	1.2	3.6	3.5	0.0	7.8	6.9	2.42
Tamil Nadu	99.3	99.6	14.6	0.8	4.2	5.6	0.0	1.4	1.2	6.24
Telangana	95.6	93.2	17.8	1.2	7.6	7.7	0.4	0.9	0.7	3.17
Tripura	99.4	96.3	16.7	1.3	4.5	6.5	0.0	2.5	2.1	5.53
Uttar Pradesh	99.5	99.3	12.7	0.6	2.3	2.9	0.2	1.6	1.2	5.02
Uttarakhand	99.4	98.3	17.0	0.9	3.6	6.2	0.3	3.2	2.6	3.38
West Bengal	99.6	97.2	13.0	0.8	3.8	4.4	0.1	0.8	0.8	6.16

HAZ = Height-for-Age Z-score; WHZ = Weight-for-Height Z-score; SD = Standard Deviation.

**Table 3:** Summary statistics of Data quality indicators for HAZ and WHZ for states of India, NFHS-5, 2019–2021.

State	Completeness of Date of Birth, %	Completeness of Anthropometry Measurement, %	Digit Preference for Height, Index of Dissimilarity, %	Digit Preference for Age at 6-Mo Intervals, Index of Dissimilarity, %	Biologically Implausible (“Flagged”) Values for HAZ, %	Biologically Implausible (“Flagged”) Values for WHZ, %	Absolute Difference in Mean HAZ by Month of Birth (December vs. January), of HAZ, *Z* Score	SD of HAZ, Z Score	SD of WHZ, *Z* Score	Height Inappropriately Measured
Andaman & Nicobar Islands	100.0	99.4	18.0	6.5	2.5	5.0	0.2	5.9	4.9	4.4
Andhra Pradesh	100.0	95.2	14.4	2.3	1.5	2.7	0.1	1.0	0.8	5.2
Arunachal Pradesh	99.9	98.3	14.8	2.0	2.8	5.8	0.2	11.5	9.6	5.9
Assam	100.0	97.7	17.1	0.3	2.8	5.8	0.3	2.6	2.2	5.5
Bihar	99.9	96.3	14.9	0.8	2.2	3.6	0.2	1.6	1.2	7.8
Chhattisgarh	100.0	96.7	14.7	1.5	2.2	4.3	0.5	2.3	1.9	4.4
Dadra & Nagar Haveli	100.0	97.0	19.2	0.9	2.0	2.7	0.5	5.1	3.8	5.7
Goa	100.0	94.4	11.4	1.7	1.4	2.4	0.6	2.5	1.9	4.4
Gujarat	99.9	98.0	16.1	1.0	2.5	5.2	0.2	2.0	1.6	5.7
Haryana	100.0	95.0	15.4	1.6	1.5	2.2	0.3	2.1	1.7	4.0
Himachal Pradesh	100.0	97.5	17.8	2.0	1.8	4.1	0.0	3.1	2.7	3.1
Jammu & Kashmir	100.0	97.8	17.0	2.3	4.4	8.1	0.7	4.2	3.6	4.2
Jharkhand	100.0	97.1	18.2	1.9	2.5	4.7	0.3	2.2	1.8	5.0
Karnataka	99.9	95.5	14.8	1.1	3.1	6.1	0.2	1.8	1.4	7.5
Kerala	100.0	96.8	11.9	2.2	1.6	3.7	0.4	1.3	1.1	5.1
Ladakh	100.0	99.8	17.9	2.0	5.6	9.2	0.6	10.1	8.6	3.3
Lakshadweep	100.0	97.8	23.9	1.3	1.8	8.8	0.4	9.0	10.1	3.2
Madhya Pradesh	99.9	94.5	15.1	0.8	1.7	3.2	0.4	1.9	1.5	4.6
Maharashtra	99.9	96.1	15.7	0.4	2.8	5.2	0.3	1.4	1.1	6.6
Manipur	100.0	99.0	13.2	3.0	1.7	2.0	0.4	4.4	3.4	4.9
Meghalaya	99.9	98.1	16.4	1.7	1.6	2.9	0.5	5.0	3.9	4.7
Mizoram	99.8	97.6	12.9	2.2	2.9	3.7	0.2	7.3	6.0	5.0
Nagaland	100.0	99.3	15.3	2.2	1.6	3.5	0.9	7.8	5.9	3.7
NCT Of Delhi	100.0	92.7	17.0	1.2	1.3	3.2	0.4	1.8	1.4	3.4
Odisha	100.0	98.0	12.8	1.6	1.2	3.2	0.3	1.8	1.6	3.9
Puducherry	100.0	99.0	19.1	4.5	1.5	2.1	0.6	3.8	3.3	7.0
Punjab	99.9	95.4	15.8	2.5	1.4	1.9	0.2	1.9	1.7	4.4
Rajasthan	99.7	97.7	11.3	1.5	2.3	4.3	0.3	1.8	1.4	4.8
Sikkim	100.0	86.9	17.1	7.2	2.0	12.1	0.6	7.2	5.9	6.4
Tamil Nadu	100.0	97.6	14.7	1.6	2.3	4.2	0.2	1.3	1.1	5.5
Telangana	100.0	93.3	15.7	1.2	3.4	6.5	0.5	2.4	1.8	7.2
Tripura	99.8	98.8	18.3	2.8	2.2	5.7	0.5	3.6	3.2	4.6
Uttar Pradesh	99.9	96.5	14.5	1.1	2.3	4.2	0.4	1.7	1.3	5.2
Uttarakhand	99.9	92.2	17.1	1.8	1.3	2.2	0.1	2.6	2.1	4.5
West Bengal	100.0	98.1	14.8	2.5	2.4	4.1	0.0	1.1	0.9	4.9

HAZ = Height-for-Age Z-score; WHZ = Weight-for-Height Z-score; SD = Standard Deviation.

## Data Availability

The International Institute for Population Sciences, Mumbai is the nodal agency for conducting NFHS-5. Data used in this study is publicly available and can be accessed from the DHS program website (https://dhsprogram.com/methodology/survey/survey-display-355.cfm, accessed on 1 November 2023) and https://www.nfhsiips.in/nfhsuser/index.php (accessed on 1 November 2023).
